# Hypercholesterolemia induced cerebral small vessel disease

**DOI:** 10.1371/journal.pone.0182822

**Published:** 2017-08-10

**Authors:** Peter Kraft, Michael K. Schuhmann, Cornelia Garz, Solveig Jandke, Daniela Urlaub, Stine Mencl, Alma Zernecke, Hans-Jochen Heinze, Roxana O. Carare, Christoph Kleinschnitz, Stefanie Schreiber

**Affiliations:** 1 Department of Neurology, University Hospital Würzburg, Würzburg, Germany; 2 Comprehensive Heart Failure Center, University of Würzburg, Würzburg, Germany; 3 Klinikum Main-Spessart, Lohr, Germany; 4 German Center for Neurodegenerative Diseases (DZNE), Magdeburg, Germany; 5 Department of Neurology, Otto-von-Guericke University, Magdeburg, Germany; 6 Department of Neurology, University Hospital Essen, Essen, Germany; 7 Institute of Clinical Biochemistry and Pathobiochemistry, University Hospital Würzburg, Würzburg, Germany; 8 Faculty of Medicine, University of Southampton, Southampton, United Kingdom; University of Münster, GERMANY

## Abstract

**Background:**

While hypercholesterolemia plays a causative role for the development of ischemic stroke in large vessels, its significance for cerebral small vessel disease (CSVD) remains unclear. We thus aimed to understand the detailed relationship between hypercholesterolemia and CSVD using the well described *Ldlr*^*-/-*^ mouse model.

**Methods:**

We used *Ldlr*^*-/-*^ mice (n = 16) and wild-type (WT) mice (n = 15) at the age of 6 and 12 months. *Ldlr*^*-/-*^ mice develop high plasma cholesterol levels following a high fat diet. We analyzed cerebral capillaries and arterioles for intravascular erythrocyte accumulations, thrombotic vessel occlusions, blood-brain barrier (BBB) dysfunction and microbleeds.

**Results:**

We found a significant increase in the number of erythrocyte stases in 6 months old *Ldlr*^*-/-*^ mice compared to all other groups (*P* < 0.05). *Ldlr*^*-/-*^ animals aged 12 months showed the highest number of thrombotic occlusions while in WT animals hardly any occlusions could be observed (*P* < 0.001). Compared to WT mice, *Ldlr*^*-/-*^ mice did not display significant gray matter BBB breakdown. Microhemorrhages were observed in one *Ldlr*^*-/-*^ mouse that was 6 months old. Results did not differ when considering subcortical and cortical regions.

**Conclusions:**

In *Ldlr*^*-/-*^ mice, hypercholesterolemia is related to a thrombotic CSVD phenotype, which is different from hypertension-related CSVD that associates with a hemorrhagic CSVD phenotype. Our data demonstrate a relationship between hypercholesterolemia and the development of CSVD. *Ldlr*^*-/-*^ mice appear to be an adequate animal model for research into CSVD.

## Introduction

In Western societies hypercholesterolemia is very common and affects 40–80% of all patients with typical macroangiopathic cardiovascular diseases comprising ischemic stroke, myocardial infarction and peripheral arterial disease [1]. Despite improved awareness of this risk factor and the increasing use of pharmacological treatments to lower cholesterol levels, preventing atherosclerosis of the large arteries still remains one of the major challenges when aiming to reduce the incidence of middle cerebral artery infarctions [2,3]. However, there are controversies about the causative role of hypercholesterolemia for microangiopathic cerebral ischemia development, which has been attributed mainly to arterial hypertension [4].

The pathological changes of small arteries and capillaries are defined as cerebral small vessel disease (CSVD), characterized by age-dependent blood-brain barrier (BBB) breakdown and arteriolosclerosis leading to small vessel occlusions, microbleeds and lacunar infarctions [5–7]. Recent human cohort studies using magnetic resonance imaging (MRI) challenged the specific associations between lacunar infarct development and hypercholesterolemia [8–10]. Furthermore, other studies claimed protective effects of hypercholesterolemia preventing from CSVD-related downstream microangiopathic lesions [11–12].

There are several mechanisms linking hypercholesterolemia with pathological features of the vessel walls potentially associated with intraluminal accumulations of cells [13], impairment of the vasodilatory response [14] and a severe inflammatory and thrombogenic phenotype [15]. There is a danger that the role hypercholesterolemia might play in inducing cerebral microangiopathy will be neglected when simply taking into account clinical MRI studies limited to visualize CSVD-related downstream pathology but not the small vasculature itself.

Using *Ldlr*^*-/-*^ mice that develop elevated plasma cholesterol levels when fed a high cholesterol diet, this study tests the hypothesis that hypercholesterolemia results in pathological changes of the walls of small vessels and in thrombosis [16]. We aim to compare the changes induced by hypercholesterolemia with arterial hypertension-induced CSVD found in spontaneously hypertensive stroke prone rats (SHRSP) [7].

## Materials and methods

### Animals

All animal experiments were approved by local state authorities (Regierung von Unterfranken, reference number 90/12) and performed in accordance with Animal Research: Reporting In Vivo Experiments (ARRIVE) guidelines (https://www.nc3rs.org.uk/arrive-guidelines). In this study male C57BL/6 wild-type (WT) mice (n = 15; 6 mice 6 months old, 9 mice 12 months old) and transgenic mice lacking the low-density lipoprotein (LDL) receptor (LDLR) (*Ldlr*^*-/-*^ mice, n = 16; 8 mice 6 months old, 8 mice 12 months old) [17] were investigated. For induction of hypercholesterolemia at 8 weeks of age, *Ldlr*^−/−^ mice were put on an atherogenic diet (15% milk fat and 1.25% cholesterol; Altromin, Germany) for 9 weeks [18]. Animals were housed with a natural light-dark cycle and had access to food and water ad libitum. Mortality was 0% in all groups, i.e. no animal died during the follow-up phase of up to 12 months.

### Histology

Mice were transcardially perfused with 120 mL phosphate buffered saline (PBS) to remove blood completely, followed by 120 mL of 4% paraformaldehyde (PFA) within 4 minutes. The rodents’ brains were removed, perfused with 4% PFA for 48 hours, placed for cryo-protection into 30% sucrose for 6 days, and frozen in methylbutane at -80°C. Coronal slices of the whole brain were prepared using a cryotome (Leica CM 1950, Wetzlar, Germany). Staining with Hematoxylin–Eosin (HE) was performed in all animals. From the frontal to the occipital pole, there were 9 sectional planes per animal, whereas the first sectional plane was a remote 1.5 mm from the frontal pole. The distance between each sectional plane was 1 mm. Three slices per brain sectional plane were stained, and thus around 27 slices per animal were available for analysis.

For all *Ldlr*^*-/-*^ and the age matched wild-type control group, the frequency of (i) small vessels with incomplete/partial occlusions due to intraluminal erythrocyte accumulations (“erythrocyte stases”), (ii) small vessels with complete occlusions due to fibrin thrombi defined by intraluminal homogeneous eosinophilic material and (iii) perivascular deposits of a diameter under 150 μm consisting of erythrocytes and hemosiderin surrounding the small vessel walls (“microbleeds”) was assessed. All changes were quantified within HE slices (n = 27 per animal) separately taking into account different brain regions (hippocampus, striatum, thalamus, corpus callosum, cortex) and small vessel types (capillaries defined through a luminal diameter < 10 μm, arterioles defined through a luminal diameter > 10 μm) [19]. The number of small vessels displaying phenomena (i), (ii) or (iii) was counted per field of view (FOV) investigating 20 to 25 FOVs per brain region in each animal.

### Immunohistochemistry

Immunoglobulin G (IgG) immunohistochemistry was performed to detect BBB breakdown as indicated by vessel wall-adherent and perivascular IgG accumulations. In short, repeated washing of the sections in PBS, blocking with 0.1 mol/L PBS, 0.5% TritonX and 10% donkey serum (Sigma, St Louis, MO, USA) was followed by staining with an anti-solanum tuberosum lectin (STL, Vector Laboratories, Burlingame, CA, USA, dilution 1:500) antibody (primary) overnight at 4°C (in PBS containing 5% donkey serum). Solanum tuberosum lectin is a fluorescein-labeled potato lectin (STL-FITC) [20] binding glycoproteins at the endothelial surface [21]. Slices were then washed anew before Cy3-donkey anti-mouse IgG (dilution 1:500) was applied as a secondary antibody for IgG detection within and surround the small vessel walls (for 2 hours at room temperature). Subsequently, nuclear DAPI staining was performed for 20 minutes at room temperature. After increasing concentrations of alcohol, slices were mounted with Histomount.

Assessment of BBB damage took place by counting all STL positive and IgG positive small vessels per FOV to calculate the percentage of IgG positive vessels. Per animal 5 slices and 10 FOVs per region (hippocampus, striatum, thalamus, corpus callosum, cortex) were analyzed. Capillaries and arterioles were considered separately (see above).

### Statistics

Data are expressed as mean ± standard deviation (SD). For statistical analysis, PrismGraph 4.0 software package (La Jolla, CA, USA) was used. Using the Kolmogorov Smirnov test Gaussian distribution was confirmed for the number of small vessels with stases and fibrin thrombi as well as for the number of small vessels accumulating IgG. To compare the *Ldlr*^*-/-*^ and the control group, as well as the different ages (6 months vs. 12 months), Analysis of Variance (ANOVA) calculation was performed with group as independent variable. The respective dependent variable was the small vessel number with stases, fibrin thrombi or IgG accumulations. Analysis was conducted for the whole brain as well as for each cerebral subregion separately. Statistical models were Bonferroni adjusted to account for the issue of multiple comparisons. Due to the low number of events, no statistical evaluation was possible for the assessment of microbleeds. *P*-values < 0.05 were considered to be statistically significant.

## Results

### Erythrocyte stases

**Fig 1:**
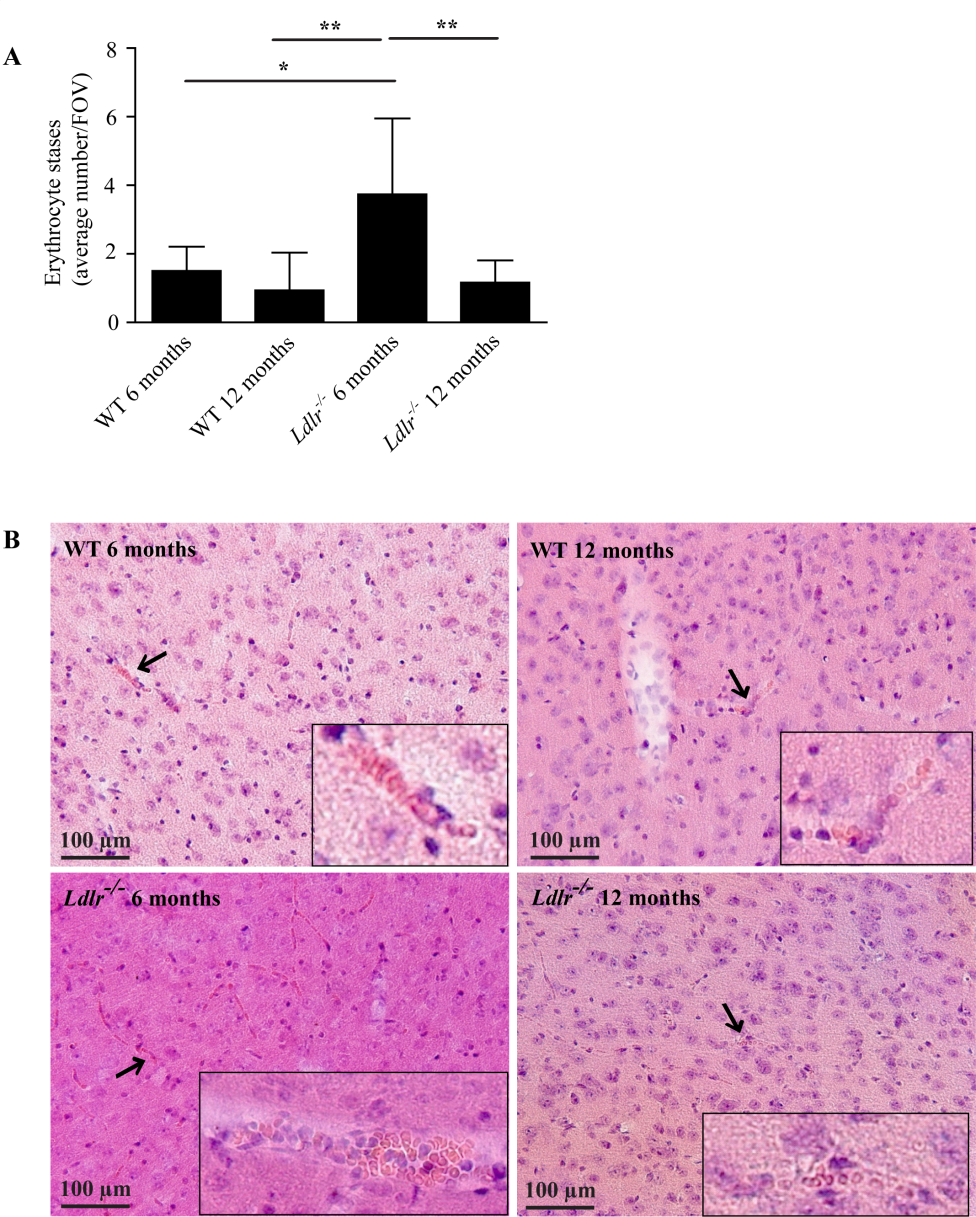
A) Intravascular accumulation of erythrocytes (referred to as erythrocyte stases) in the capillaries and arterioles of 6 and 12 months old WT and *Ldlr*^*-/-*^ mice. Importantly, 6 months old *Ldlr*^*-/-*^ animals showed the highest number of stases. Analysis of variance with Bonferroni post-hoc test, * *P* < 0.05, *P* < 0.01. B) Histological Hematoxylin–Eosin stainings of cortical tissue of 6 and 12 months old WT and *Ldlr*^*-/-*^ mice (20x magnification). Erythrocyte accumulations are marked with arrows and accentuated in the inserts with higher magnification (40x). WT, wild-type; FOV, field of view. Error bars indicate standard deviation.

In order to evaluate structural changes induced by hypercholesterolemia in the walls of small vessels, we investigated the brains from 6 and 12 months old *Ldlr*^*-/-*^ mice fed a high fat diet for 9 weeks in comparison to age-matched WT mice fed a normal chow. As a first step we histologically assessed HE stained brain slices for incomplete small vessel occlusions induced by the accumulation of erythrocytes, also referred to as erythrocyte stases. Within the evaluation of all capillaries and arterioles in the different cerebral regions examined, we found 1.5 ± 0.7 erythrocyte stases per FOV in the group of 6 months old WT animals (**Fig 1A and 1B**). Age had no direct effect on intravascular erythrocyte accumulation, as 12 months old WT animals seemed to have fewer stases although this did not achieve statistical significance (0.9 ± 1.1/FOV, *P* > 0.05). In contrast, 6 months old *Ldlr*^*-/-*^ mice showed a significant increase of stases (3.7 ± 2.2/FOV) compared to both WT groups (*P* < 0.05 compared to WT 6 months old, *P* < 0.01 compared to WT 12 months old). Surprisingly, we could not find an elevated level of erythrocyte accumulations in the aged *Ldlr*^*-/-*^ mice (1.2 ± 0.6/FOV; *P* < 0.01 compared to *Ldlr*^*-/-*^ 6 months old, *P* > 0.05 compared to the WT groups) (**Fig 1A and 1B**).

Our detailed histological examination allows an in-depth subgroup analysis for each brain region and vessel type. The number of stases varied between regions (e.g. 2.2 ± 1.3/FOV in the cortex of WT mice 6 months old *vs*. 0.4 ± 0.2/FOV in the corpus callosum of WT animals 6 months old) but the general pattern observed in the compound analysis (**Fig 1A**) was maintained (**S1 Fig**). Regarding histological analyses of different vessel types we found more erythrocyte stases in capillaries compared to arterioles (e.g. 3.7 ± 2.2/FOV in the capillaries of *Ldlr*^*-/-*^ mice 6 months old *vs*. 0.1 ± 0.0/FOV in the arterioles of *Ldlr*^*-/-*^ mice 6 months old). In contrast to the pattern in Fig 1A and Supplemental Figure I, there was a borderline significance for the increase of arteriolar but not capillary stases in 12 months old *Ldlr*^*-/-*^ animals compared to WT mice (*P* = 0.05 for both age groups) (**S2 Fig**).

### Fibrin thrombi

**Fig 2:**
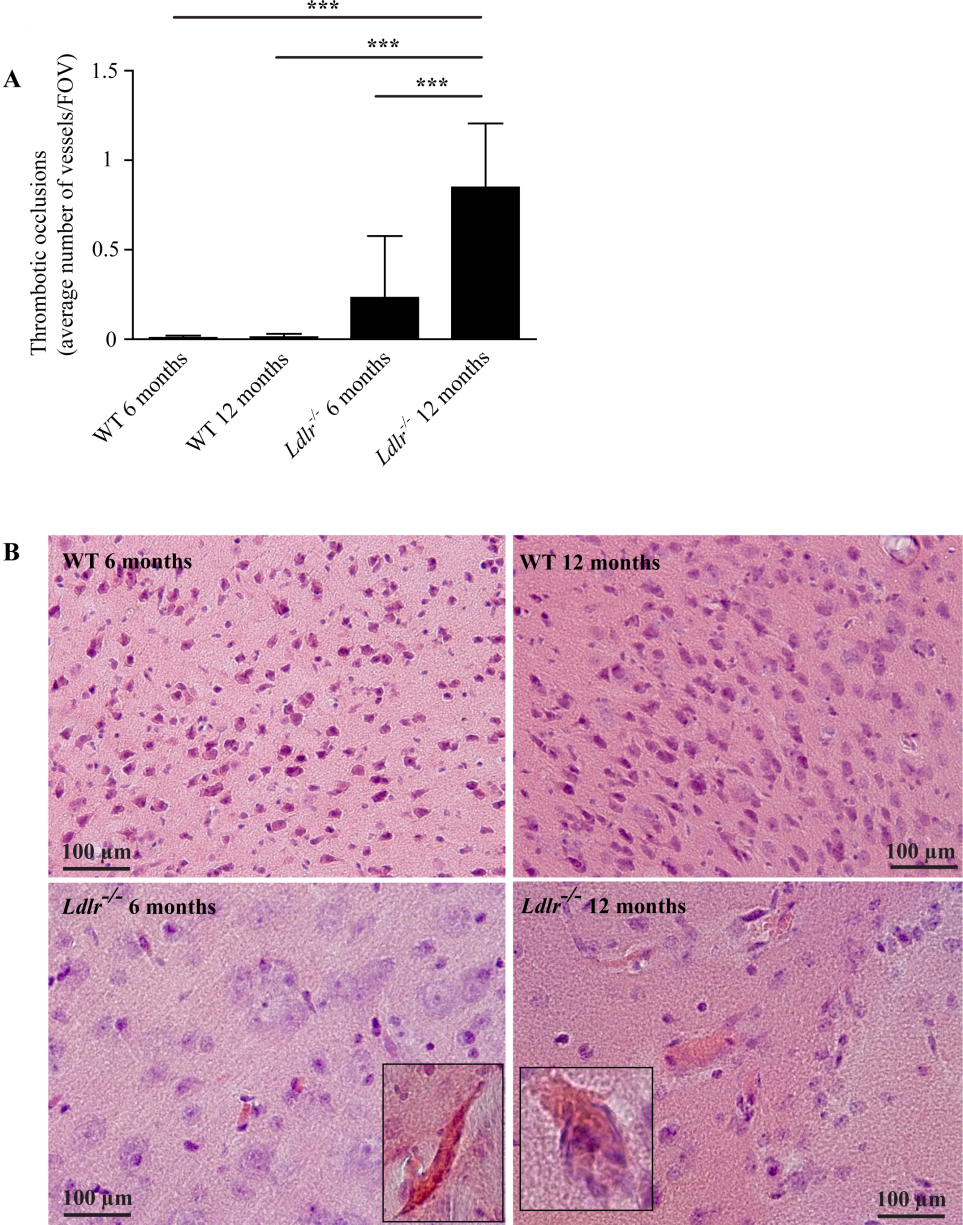
A) Thrombotic occlusions (referred to as suspected fibrin thrombi) of capillaries and arterioles of 6 and 12 months old WT and *Ldlr*^*-/-*^ mice. 12 months old *Ldlr*^*-/-*^ animals displayed more occlusions than any other group. Analysis of variance with Bonferroni post-hoc test, *** *P* < 0.001. B) Histological Hematoxylin–Eosin stainings of cortical (WT 6 and 12 months, *Ldlr*^*-/-*^ 6 months) and hippocampal tissue (*Ldlr*^*-/-*^ 12 months) (20x magnification). Vessel occlusions are highlighted using larger magnification (40x). WT, wild-type; FOV, field of view. Error bars indicate standard deviation.

As erythrocyte stases may represent an early step of CSVD in the older age group [7], we hypothesized that 12 months old mice with hypercholesterolemia would have an increased number of erythrocyte accumulations compared to younger animals. The data demonstrate that the number of stases was lower in 12 months compared to 6 months old *Ldlr*^*-/-*^ mice. In the compound analysis we found almost no occluded vessels in the WT animals (0.01 ± 0.02/FOV in 12 months old WT mice) and only moderate numbers in 6 months old *Ldlr*^*-/-*^ mice (0.23 ± 0.35/FOV) but a dramatic increase in the 12 months *Ldlr*^*-/-*^ group (0.85 ± 0.36/FOV, *P* < 0.001 compared to all other groups) (**Fig 2A and 2B**).

In a subgroup analysis we found the same pattern in all investigated brain areas with differences in the absolute number of vessel occlusions (e.g. thrombotic occlusions in 12 months old *Ldlr*^*-/-*^ mice: 2.0 ± 1.0/FOV (cortex) vs. 0.2 ± 0.3/FOV (corpus callosum)) (**S3 Fig**). We found fewer thrombi in arterioles compared to capillaries (e.g. thrombotic occlusions in 12 months old *Ldlr*^*-/-*^ mice: 0.8 ± 0.3/FOV (capillary) *vs*. 0.1 ± 0.1/FOV (arteriolar)) (**S4 Fig**), but the general pattern remained preserved compared to the compound analysis (**Fig 2**).

### Blood-brain barrier leakage

**Fig 3:**
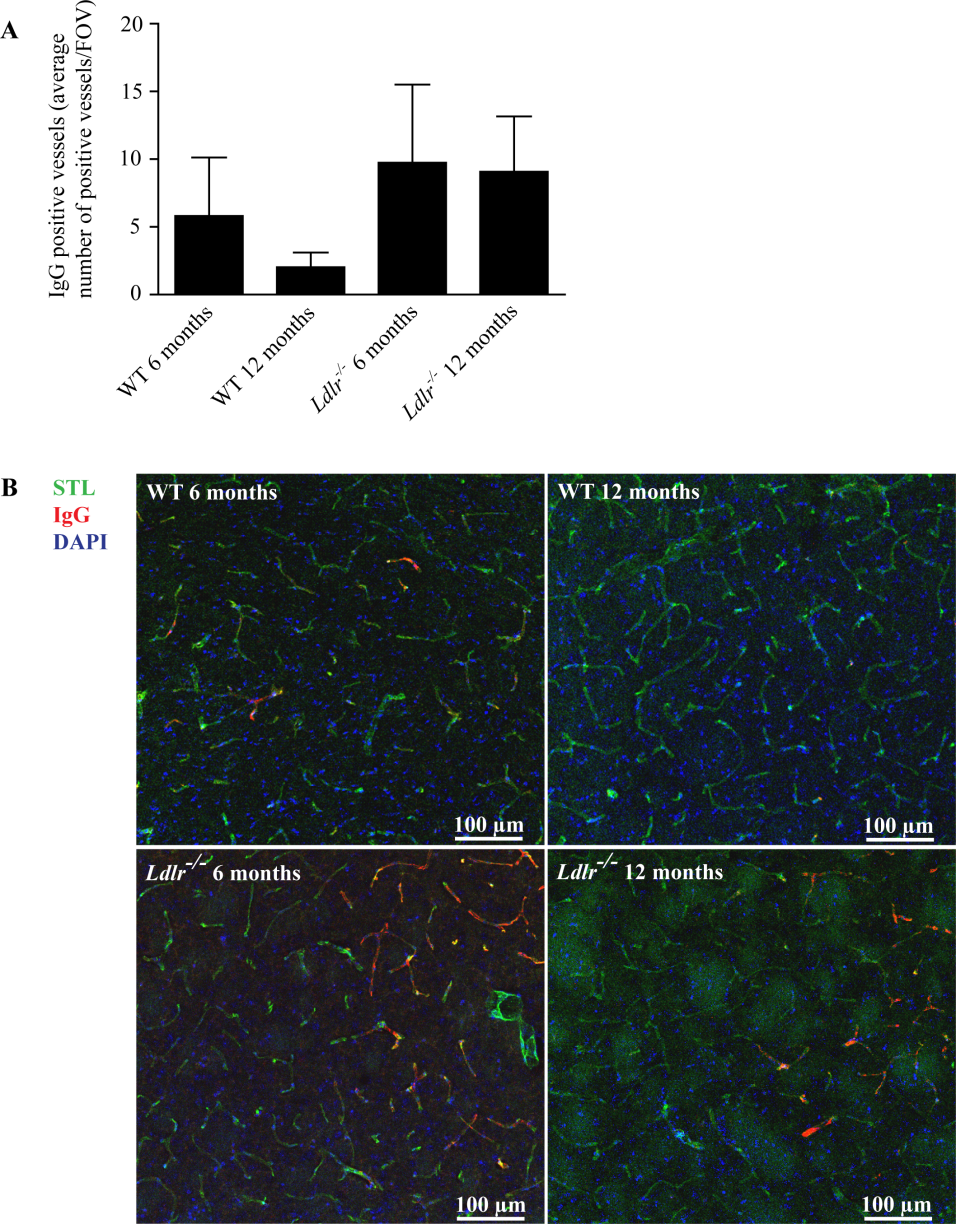
A) IgG positive capillaries and arterioles of 6 and 12 months old WT and *Ldlr*^*-/-*^ mice. No significant difference could be observed between the groups (analysis of variance with Bonferroni post-hoc test, *P* > 0.05). B) Immunohistochemical stainings for STL (green), IgG (red) and DAPI (blue) of brain tissue of 6 and 12 months old WT and *Ldlr*^*-/-*^ mice (20x magnification, WT 6 months old: thalamus, WT 12 months old and *Ldlr*^*-/-*^ mice: striatum). WT, wild-type; FOV, field of view; STL, solanum tuberosum lectin (endothelial marker); IgG, immunoglobulin G; DAPI, 4′.6-diamidino-2-phenylindole (nuclear staining). Error bars indicate standard deviation.

As recent publications point towards disturbances of the blood-brain barrier (BBB) in CSVD [5, 22], we determined the occurrence of IgG deposits in and around the walls of small vessels in the hippocampus, the striatum, the thalamus, the cortex, and the corpus callosum as a sign of BBB leakage [7]. In contrast to erythrocyte stases and fibrin thrombi, we found no significant differences in IgG positive vessels between WT and *Ldlr*^*-/-*^ mice (*P* > 0.05) in the compound analysis. Age had no effect on the number of IgG deposits (P > 0.05) (**Fig 3**).

In the subgroup analysis regarding the different brain regions there was no gross difference in the number of IgG positive vessels with the exception of the corpus callosum where we found a significant increase in the 12 months old *Ldlr*^*-/-*^ group (14.3 ± 6.4/FOV) compared to 6 months (4.9 ± 1.1/FOV, *P* < 0.05) and 12 months old WT mice (2.1 ± 0.6/FOV, *P* < 0.01) (**S5 Fig**). Arterioles and capillaries of *Ldlr*^*-/-*^ animals showed slightly increased numbers of IgG positive vessels compared to WT animals but only the comparison of arterioles between 12 months old WT (0.2 ± 0.1/FOV) and *Ldlr*^*-/-*^ mice (0.8 ± 0.3/FOV) yielded a significant difference (*P* < 0.05) (**S6 Fig**).

### Microbleeds

Based on the observation that cerebral microbleeds are common in a rat model of hypertension-related CSVD [7] we aimed to assess the frequency and localization of microbleeds in *Ldlr*^*-/-*^ mice. Hardly any microbleeds could be detected in the present study. From all animals examined, we found only 1 microbleed located in the cortex of a 6 month old *Ldlr*^*-/-*^ mouse.

## Discussion

In the present study we identified that *Ldlr*^*-/-*^ mice fed a high fat diet–a well-known transgenic mouse model of hypercholesterolemia–develop key features of cerebral capillary and arteriolar degeneration. While erythrocyte stases were more abundant in younger mice only, advanced small vessel pathology, i.e. fibrin thrombi, was primarily found in older animals. We assume that erythrocyte stases could also be observed in 12 months year old *Ldlr*^*-/-*^ mice if the microvessels were still open. Therefore, the high number of thrombotic vessel occlusions in the old *Ldlr*^*-/-*^ mice fully explains the finding of reduced erythrocyte stases in 12 months compared to 6 months old *Ldlr*^*-/-*^ animals. Based on these results, we postulate a cascade of age-dependent histological changes that occur towards the development of hyperlipidemia-related CSVD. In a first step erythrocyte stasis can be observed, in a second step (time-dependent) thrombotic occlusions of microvessels occure with the consequence of reduced erythrocyte stasis.

In hypercholesterolemic mice, BBB damage was confined to the white matter, while there were no vascular IgG deposits or microbleeds observed in the gray matter of the *Ldlr*^*-/-*^ model.

To the best of our knowledge, our in-depth histological assessment is the first study on microvascular pathology indicating CSVD in hypercholesterolemic *Ldlr*^*-/-*^ brains. Our data add important value to the controversies in the field by suggesting a causative role of hypercholesterolemia for microvascular damage initiation.

We have previously identified a cascade of age-dependent histological changes that occur towards the development of hypertension-related CSVD in the SHRSP rat model. That cascade is initiated by microvascular endothelial damage as indicated by BBB-breakdown, erythrocyte stases and cerebral blood flow reductions identified early in young rats [7]. At later stages and higher age groups, increasing fragility of the walls of small vessels leads to perivascular microbleeds and reactive fibrin thromboses. Our present results derived from *Ldlr*^*-/-*^ mice display similarities and differences between the two rodent strains used for the study of hypercholesterolemia-induced CSVD on the one hand and hypertension-related small vessel wall damage on the other hand. Firstly, erythrocyte stases as a proxy of endothelial-activation related wall-adherent clustering of blood cells seems to be the starting point of small vessel wall damage independently from the CSVD causing vascular risk factor. Secondly, fibrin thromboses have to be considered as a final CSVD stage indicating ongoing and progressive clumping of blood cells accompanied by endothelial inflammation, reflecting an acceleration of the coagulation cascade. Key differences between the two rodent models comprise that hypertension-induced CSVD is characterized by widespread BBB dysfunction affecting cortical and subcortical regions accompanied by a remarkable number of cerebral microbleeds [7], whereas *Lldr*^*-/-*^ mice display minimal BBB damage restricted to the white matter and no microbleeds. Our results suggest that small vessel occlusions are induced by hypertension as well as hypercholesterolemia, but small vessel wall rupture occurs more frequently as a result of hypertension.

*Ldlr*^*-/-*^ mice were initially developed as a mouse model of familial hypercholesterolemia caused by mutations in the gene for the LDL receptor. When fed a high fat diet, these mice develop macroangiopathy and atherosclerosis, starting from the proximal aorta. While in *Ldlr*^*-/-*^ mice there is a well-established link between high serum cholesterol levels and macroangiopathic lesions [16, 17], the pathological mechanism of hypercholesterolemia in the cerebral microcirculation has not been addressed yet. Hypercholesterolemia induces the upregulation of endothelial proteins such as P-Selectin [13], that, together with elevated blood viscosity [23], increased neutrophilic shear-sensitivity within the small vessel bed [24] and autoregulatory alterations [14] result in a microvascular inflammatory-thrombotic state. These changes may also be responsible for the small vessel occlusions found in our study. *Ldlr*^*-/-*^ mice may also develop endothelial disturbances due to the LDL receptor deficiency itself. This assumption can be corroborated by data showing an increased inactivation of H_2_O_2_ (an endothelial relaxation factor in *Ldlr*^*-/-*^ mice) [25, 26] and by the fact that the LDL receptor serves as a binding site for the blood coagulation factor VIII, thus mediating its clearance from the circulation [27]. Taken together, it is very likely that hypercholesterolemia-dependent and -independent mechanisms contribute to CSVD development in *Ldlr*^*-/-*^ mice.

There were nearly no microhemorrhages in our mouse model of hypercholesterolemia. While this is contrary to the histological results gathered in the microvasculature of SHRSP [7], our findings in *Ldlr*^*-/-*^ mice are, however, in line with clinical studies showing that (i) low serum cholesterol levels are associated with an increased number of cerebral microbleeds and with ongoing hematoma growth after large intracerebral bleeding, and that (ii) statin-use is not related to intracerebral hemorrhage occurrence [14, 28–31]. We hypothesize, that this inverse relationship between hypercholesterolemia and microbleed load might be explained by hypercholesterolemia-induced small vessel degeneration which rather cause wall thickening and arteriolosclerosis. Although detrimental in nature, thickening of the vascular walls may reduce wall fragility protecting the microvasculature from rupture and thus from perivascular bleeding. Sustained microvascular wall stability is supported by the overall missing differences between WT and *Ldlr*^*-/-*^ mice with regards to BBB function, at least when considering cortical and subcortical gray matter structures alone. Circumscribed BBB breakdown found in the white matter could be an early predictor of white matter damage commonly found in CSVD [4].

Similar to our data in SHRSP, the hypercholesterolemia-induced CSVD pattern did not differ between cerebral subregions supporting that small vessel wall damage is not restricted to certain brain regions such as the basal ganglia or white matter. Similar observations were obtained from recent human MRI studies suggesting a pathological involvement of the small vasculature in several different cortical areas [5].

The strength of our study lies in the in-depth histological analysis of various brain regions, the investigation of animals at different age groups and the assessment of isolated hypercholesterolemia effects on the cerebral microvasculature independent from comorbidities such as diabetes mellitus or arterial hypertension. Moreover, *Ldlr*^*-/-*^ mice display highly elevated plasma cholesterol levels with the hypercholesterolemia being confined mainly to the LDL fraction similar to the plasma lipoprotein profile found in humans with hypercholesterolemia [16].

One limitation is that we did not perform standardized measurements of serum cholesterol levels. We thus could not relate our findings to the severity of hypercholesterolemia. We did not screen for potential large artery stenosis, although this has been reported. *Ldlr*^*-/-*^ mice develop atherosclerotic lesions primarily in the aorta and innominate artery, and after prolonged periods of diet also within the carotid arteries, in particular the external carotid artery [32]. The small vessel stases and thromboses observed in the present study were not restricted to the territory of one large brain perfusing artery but were instead present in several territories not supplied by one large vessel. We thus assume that our CSVD findings should not be based on an interaction between macroangiopathic stenoses and small vessel alterations.

## Conclusions

This study demonstrates the relation between hypercholesterolemia and a thrombotic CSVD phenotype in *Ldlr*^*-/-*^ mice, suggesting them as a suitable model for research into CSVD.

## Supporting information

S1 Fig Intravascular accumulation of erythrocytes (referred to as erythrocyte stases) in the capillaries and arterioles of 6 and 12 months old WT and *Ldlr*^*-/-*^ mice: analysis of different brain regions (cortex, striatum, hippocampus, corpus callosum, thalamus). Analysis of variance with Bonferroni post-hoc test, * *P* < 0.05, ** *P* < 0.01. WT, wild-type; Ldlr^-/-^, low-density lipoprotein receptor deficient mice.
(PDF)

S2 Fig Intravascular accumulation of erythrocytes (referred to as erythrocyte stases) in the capillaries or arterioles of 6 and 12 months old WT and *Ldlr*^*-/-*^ mice. Analysis of variance with Bonferroni post-hoc test, * *P* < 0.05, ** *P* < 0.01. WT, wild-type; Ldlr^-/-^, low-density lipoprotein receptor deficient mice; n.s., not significant.
(PDF)

S3 Fig Thrombotic occlusions (referred to as suspected fibrin thrombi) of capillaries and arterioles of 6 and 12 months old WT and *Ldlr*^*-/-*^ mice: analysis of different brain regions (cortex, striatum, hippocampus, corpus callosum, thalamus). Analysis of variance with Bonferroni post-hoc test, * *P* < 0.05, ** *P* < 0.01, *** *P* < 0.001. WT, wild-type; Ldlr^-/-^, low-density lipoprotein receptor deficient mice.
(PDF)

S4 Fig Thrombotic occlusions (referred to as suspected fibrin thrombi) of capillaries or arterioles of 6 and 12 months old WT and *Ldlr*^*-/-*^ mice. Analysis of variance with Bonferroni post-hoc test, * *P* < 0.05, *** *P* < 0.001. WT, wild-type; Ldlr^-/-^, low-density lipoprotein receptor deficient mice.
(PDF)

S5 Fig IgG positive capillaries and arterioles of 6 and 12 months old WT and *Ldlr*^*-/-*^ mice: analysis of different brain regions (cortex, striatum, hippocampus, corpus callosum, thalamus). Analysis of variance with Bonferroni post-hoc test, * *P* < 0.05, ** *P* < 0.01. WT, wild-type; Ldlr^-/-^, low-density lipoprotein receptor deficient mice; n.s., not significant.
(PDF)

S6 Fig IgG positive capillaries or arterioles of 6 and 12 months old WT and *Ldlr*^*-/-*^ mice. Analysis of variance with Bonferroni post-hoc test, * *P* < 0.05. WT, wild-type; Ldlr^-/-^, low-density lipoprotein receptor deficient mice; n.s., not significant.
(PDF)
