# Supplementary material for: Hypercholesterolemia induced cerebral small vessel disease
Source: PLoS One. 2017 Aug 10;12(8):e0182822. doi: 10.1371/journal.pone.0182822 (PMC5552130; doi:10.1371/journal.pone.0182822)
Supplement: S2 Fig — Analysis of variance with Bonferroni post-hoc test, * P < 0.05, ** P < 0.01. WT, wild-type; Ldlr-/-, low-density lipoprotein receptor deficient mice; n.s., not significant. (PDF) [file pone.0182822.s002.pdf]

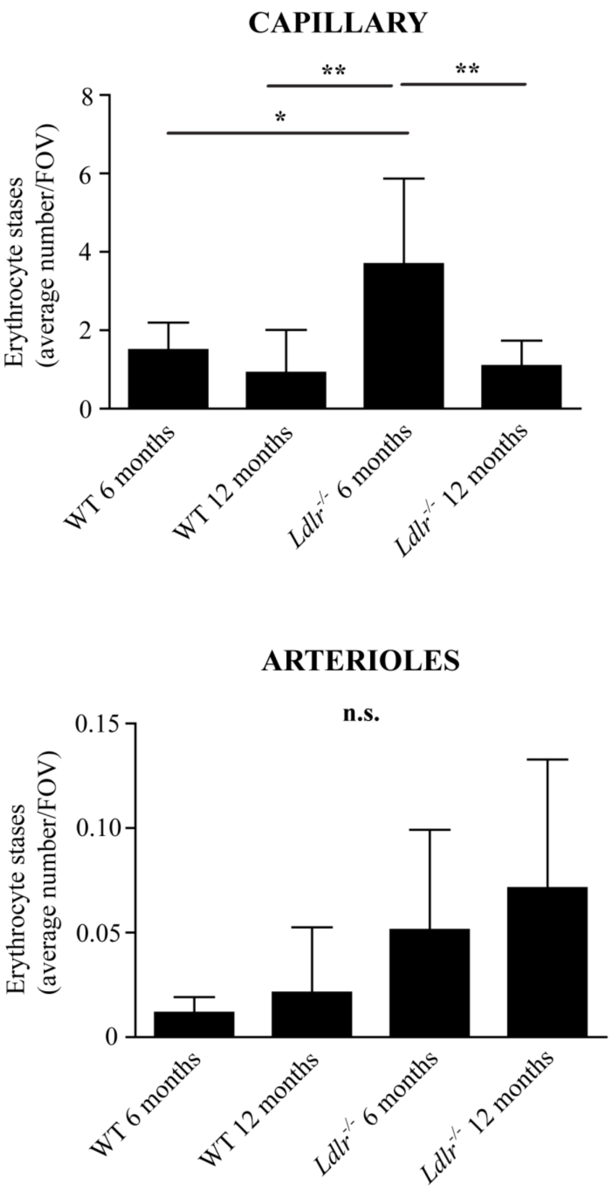

S2 Fig. Intravascular accumulation of erythrocytes (referred to as erythrocyte stases) in the capillaries or arterioles of 6 and 12 months old WT and *Ldlr*$^{-/-}$ mice. Analysis of variance with Bonferroni post-hoc test, * $P < 0.05$, ** $P < 0.01$. WT, wild-type; Ldlr$^{-/-}$, low-density lipoprotein receptor deficient mice; n.s., not significant.
