# Supplementary material for: Hypercholesterolemia induced cerebral small vessel disease
Source: PLoS One. 2017 Aug 10;12(8):e0182822. doi: 10.1371/journal.pone.0182822 (PMC5552130; doi:10.1371/journal.pone.0182822)
Supplement: S3 Fig — Analysis of variance with Bonferroni post-hoc test, * P < 0.05, ** P < 0.01, *** P < 0.001. WT, wild-type; Ldlr-/-, low-density lipoprotein receptor deficient mice. (PDF) [file pone.0182822.s003.pdf]

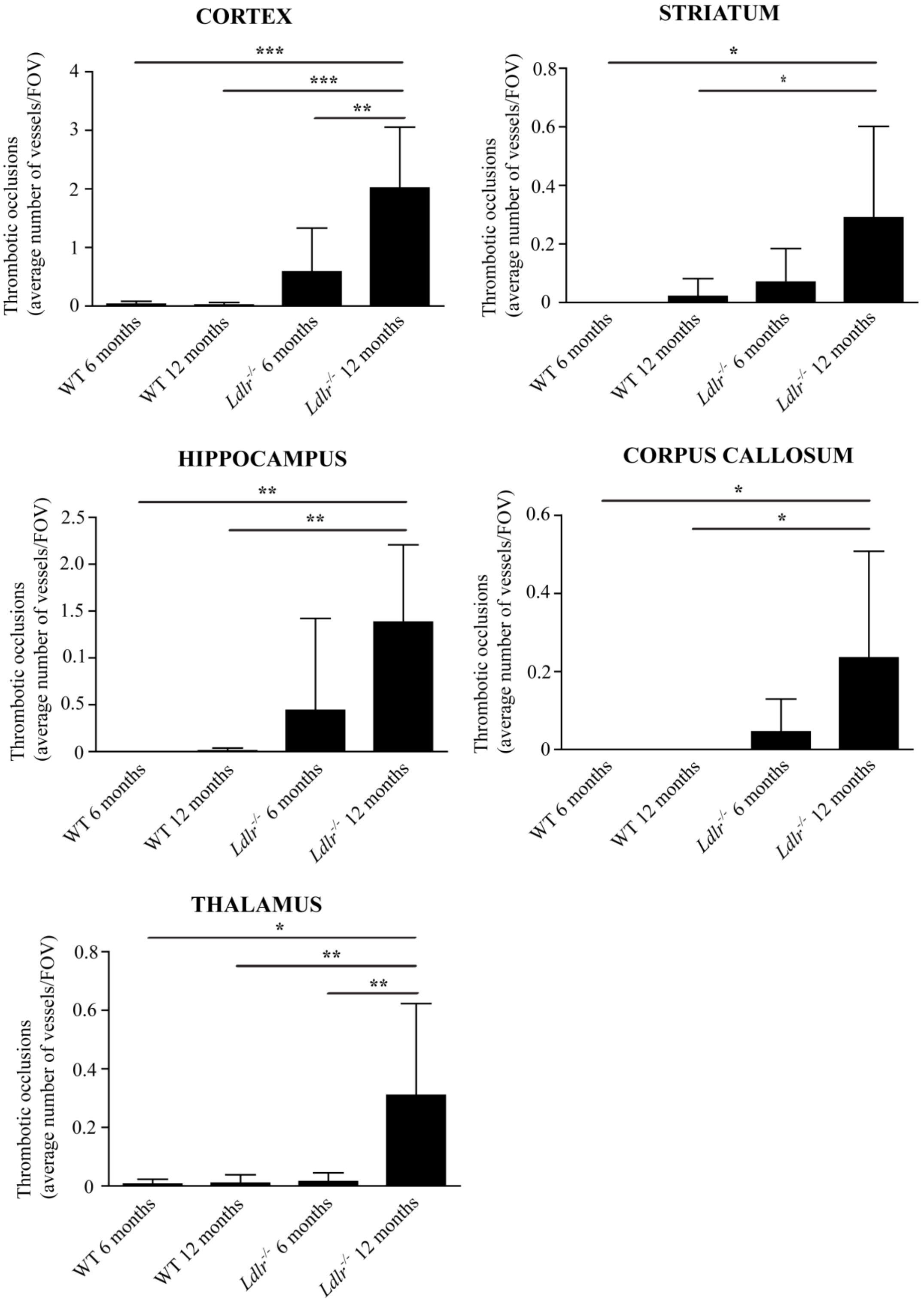

S3 Fig. Thrombotic occlusions (referred to as suspected fibrin thrombi) of capillaries and arterioles of 6 and 12 months old WT and *Ldlr*$^{-/-}$ mice: analysis of different brain regions (cortex, striatum, hippocampus, corpus callosum, thalamus). Analysis of variance with Bonferroni post-hoc test, * $P < 0.05$, ** $P < 0.01$, *** $P < 0.001$. WT, wild-type; Ldlr$^{-/-}$, low-density lipoprotein receptor deficient mice.
